# Role of microRNA miR171 in plant development

**DOI:** 10.7717/peerj.15632

**Published:** 2023-07-10

**Authors:** Ling Ling Pei, Ling Ling Zhang, Xin Liu, Jing Jiang

**Affiliations:** 1 College of Horticulture, Shenyang Agricultural University, Shenyang, Shenhe District, China; 2 College of Horticulture, Shenyang Agriculture University, Shenyang, Shenhe District, China; 3 Horticulture Department, Shenyang Agricultural University, Shenyang, Shenhe District, China

**Keywords:** miR171, GRAS, Growth and development, Environment stress

## Abstract

MicroRNAs (miRNAs) are endogenous non-coding small RNA with 19–24 nucleotides (nts) in length, which play an essential role in regulating gene expression at the post-transcriptional level. As one of the first miRNAs found in plants, miR171 is a typical class of conserved miRNAs. The miR171 sequences among different species are highly similar, and the vast majority of them have both “GAGCCG” and “CAAUAU” fragments. In addition to being involved in plant growth and development, hormone signaling and stress response, miR171 also plays multiple and important roles in plants through interactions with microbe and other small-RNAs. The miRNA functions by regulating the expression of target genes. Most of miR171’s target genes are in the GRAS gene family, but also include some NSP, miRNAs, lncRNAs, and other genes. This review is intended to summarize recent updates on miR171 regarding its function in plant life and hopefully provide new ideas for understanding miR171 function and regulatory mechanisms.

## Introduction

MicroRNAs (miRNAs) are endogenous non-coding small RNA transcripts of 19–24 nucleotides (nts) that play essential roles in the growth, development, and stress tolerance of plants and animals by regulating gene expression (Yang et al., 2017; Huang et al., 2021). Lee, Feinbaum & Ambros (1993) discovered the first members of the miRNA family in Caenorhabditis elegans in 1993. . Since then, miRNAs have been the subject of several studies.

MiRNAs are the end products of miRNA-encoding genes that undergo a series of processing processes. The genes encoding miRNA are transcribed by RNA polymerase II to form a primary transcript, pri-miRNA, which is a few hundred nts long (Lee et al., 2004). DCL1, in association with HYL1 and SE proteins, processes pri-miRNAs at the bottom of the stem to liberate pre-miRNAs (Kurihara & Watanabe, 2004; Kurihara, Takashi & Watanabe, 2006; Lobbes et al., 2006). DCL1 continues to act on pre-miRNA to form miRNA-miRNA^∗^ duplex (Kurihara & Watanabe, 2004). HEN1 then proceeds to methylate the duplex; these 3′-terminal modifications do not occur in animal miRNAs (Yang et al., 2006). The miRNA duplex binds with the cytoplasmic Argonaute (AGO) protein, leading to the removal and degradation of the miRNA* strand, ultimately forming the miRNA-induced silencing complex (miRISC) (Schwarz et al., 2003). The miRISC complex plays a crucial role in post-transcriptional gene regulation.

MiRNAs negatively regulate their target genes by cleaving complementary mRNA or inhibiting translation at the post-transcriptional level in many biological processes. Studies also demonstrate that several miRNAs mediate DNA methylation to silence genes (Huang et al., 2021; Zhang et al., 2006; Jover-Gil, Candela & Ponce, 2005). Most of the target genes of miRNAs are transcription factors involved in a wide range of plant processes, including growth and development, metabolism, abiotic and biotic stress, among others (Bernardi et al., 2022; Jiang et al., 2018b; Huang et al., 2016; Liu et al., 2018; Luis et al., 2012; Zhang et al., 2010).

The first plant miRNA in *Arabidopsis thaliana* was discovered by researchers in 2002. Reinhart et al. (2002) identified 16 miRNAs in *Arabidopsis*, including miR171 and its homologs in rice (Reinhart et al., 2002). With advancements in research methods, many plant miRNAs have been discovered and characterized. *MiR171* is a conserved 21-nt miRNA found in several plant species, such as *Arabidopsis thaliana*, *Oryza sativa*, *Solanum lycopersicum*, *Lilium pumilum*, *Hordeum vulgare*, *Morus alba*, and others (Mahale et al., 2014; Yang et al., 2017; Kravchik et al., 2019; Yan et al., 2022; Curaba et al., 2013; Sun et al., 2022). As research has progressed, the *miR171* functions have been discovered in plant growth, development, and stress responses, such as flowering time, phase transition, drought, extreme temperature, virus, and so on (Sun et al., 2022; Huang et al., 2019; Huang et al., 2016; Curaba et al., 2013; Um et al., 2022; Huang et al., 2016; Jiang et al., 2018b; Shi et al., 2022; Yan et al., 2022). In recent years, scientists have made many advances in their understanding of the miR171 family in plants, but the analysis and classification of these developments are missing. This review concentrates on the roles of miR171 and its target genes in plant life and aims to elucidate the possible functional mechanisms of miR171. The findings should be useful for researchers working on plant microRNAs, particularly the miR171 family. It may also serve as a theoretical basis for future developments in the study of other miRNAs, the study of miR171 function in other species, or even novel functional aspects of miR171.

### Survey methodology

In this article, the PubMed (https://pubmed.ncbi.nlm.nih.gov/), Web of Science (https://www.webofscience.com/wos/alldb/basic-search), Baidu Academic (https://xueshu.baidu.com/) and sci-hub (https://sci-hub.se/) to search for literature. The search keywords and their combinations included “miRNA”; “*miR171*”; “water stress”; “plant miRNA”; “GRAS Family”; “drought stress”; “plant development and growth”; “abiotic stress”; “biotic stress”; “target gene of *miR171*”; “phylogenetic tree”; “Scarecrow-like”; “small-RNA”; “*miR171* interaction with”; “*microRNA171*”; “Embryogenesis-Associated microRNAs”; “abscisic acid”; “auxin”; “miR171 and its target gene”. We collected and screened a large number of related studies based on their relevance to the topic, and excluded those unrelated. We collected and reviewed several related studies for relevance to the topic and excluded the unrelated ones. To examine the conservation and specificity of miR171 mature sequences across different species, we obtained the sequences from the PmiREN2.0 database (https://www.pmiren.com/) and carried out a sequence alignment using DNAMAN software. Moreover, we generated a maximum likelihood evolutionary tree of miR171’s mature and precursor sequences utilizing MEGA software, considering representative species for this analysis. Our goal was to describe the function of miR171 and its target genes in plant life and discuss the possible functional mechanisms of miR171. Therefore, we also excluded articles with less relevance after determining their focus by reading the abstract.

### MiR171 and its target genes in plants

#### Members of the miR171 family

**Figure 1:**
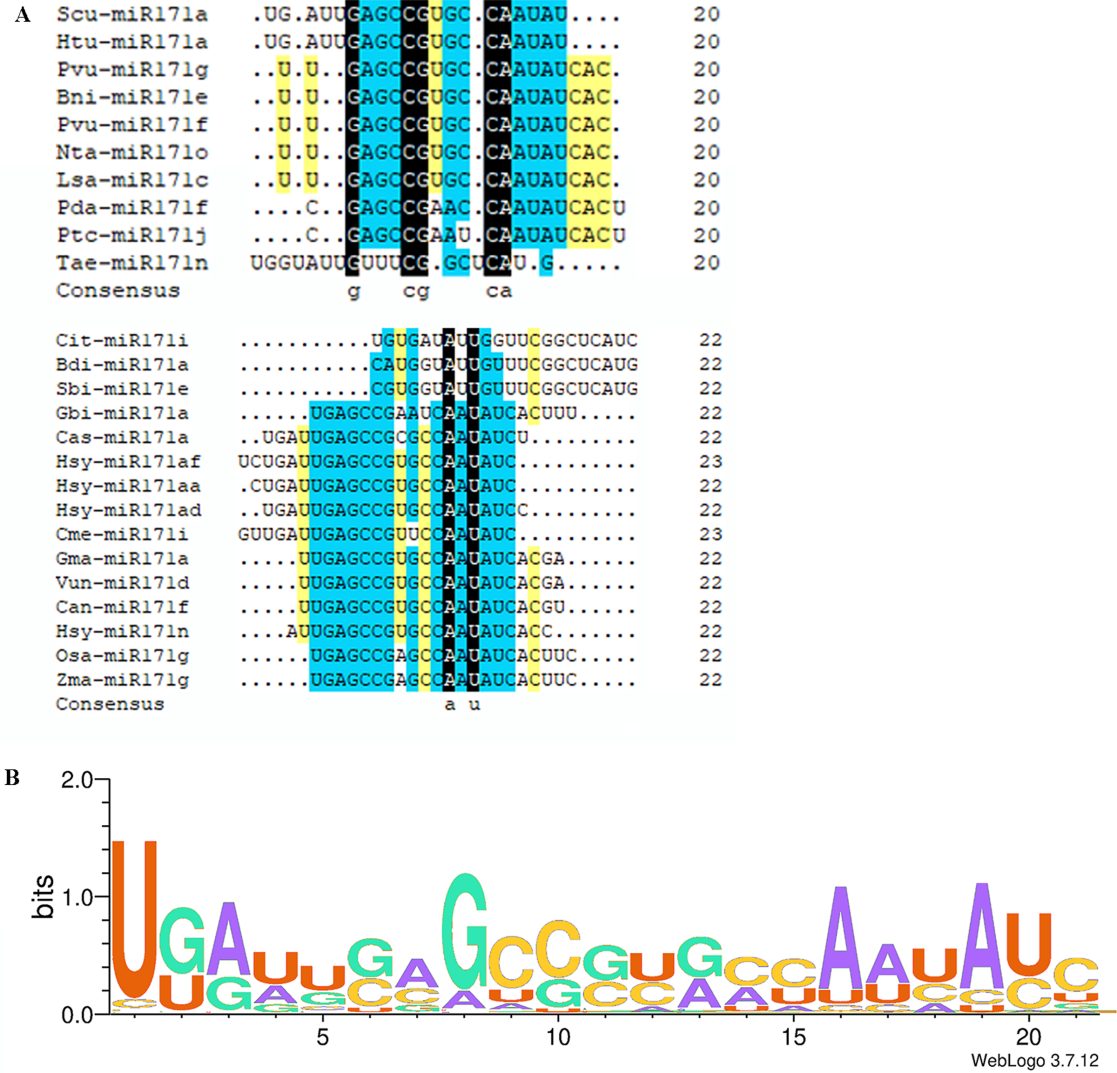
Multi sequence alignment of specially mature miR171. (A) Multiple sequence alignment of the non-21 nts mature miR171s; (B) Sequence logo showing a consensus sequence generated from multiple alignments of mature miR171s from different plant species. Black indicates a homology level of 100%, yellow indicates a homology level above 75%, and blue indicates a homology level above 50%.

The *miR171* family currently comprises eight hundred and seventeen members. The Plant MicroRNA Encyclopedia (https://www.pmiren.com/) lists their mature sequences in one hundred eight species. The *miR171* family is the second-largest miRNA family (Zhang et al., 2021a; Zhang et al., 2021b). In most plants, the members appear in the 21 nts form; however, some 20 nts, 22 nts, and 23 nts *miR171* families were also present in other species (Fig. 1A). We observed twenty-nine *miR171* members in 20 nts form in eleven species (*Bni-miR171e*, *Pda-miR171f*, *Jre-miR171a*, *Rsa-miR171a-d*, *Scu-miR171a-l*, *Tae-miR171n/o*, *Hlu-miR171b*, *Lsa-miR171c*, *Lja-miR171e-g*, *Nta-miR171o*, *Pvu-miR171f/g*); twenty-two 22 nts in seventeen species (*Bdi-miR171a*, *Can-miR171f*, *Cit-miR171i*, *Gbi-miR171a*, *Gma-miR171a/i*, *Han-miR171a*, *Hsy-miR171n/aa/ae/ad*, *Lsa-miR171e*, *Mdo-miR171b*, *Osa-miR171 g*, *Pdu-miR171a*, *Pca-miR171b/d*, *Sbi-miR171e*, *Scu-miR171a*, *Tha-miR171a*, *Vun-miR171d*, *Zma-miR171 g*); and six 23 nts in two species (*Cme-miR171i*, *Hsy-miR171a/y/af/ai/al*). Notably, both sugarcane and radish *miR171* mature sequences are 20 nts long, suggesting that they may have a closer genetic relationship and similar functional properties.

### Conservation of miR171

**Figure 2:**
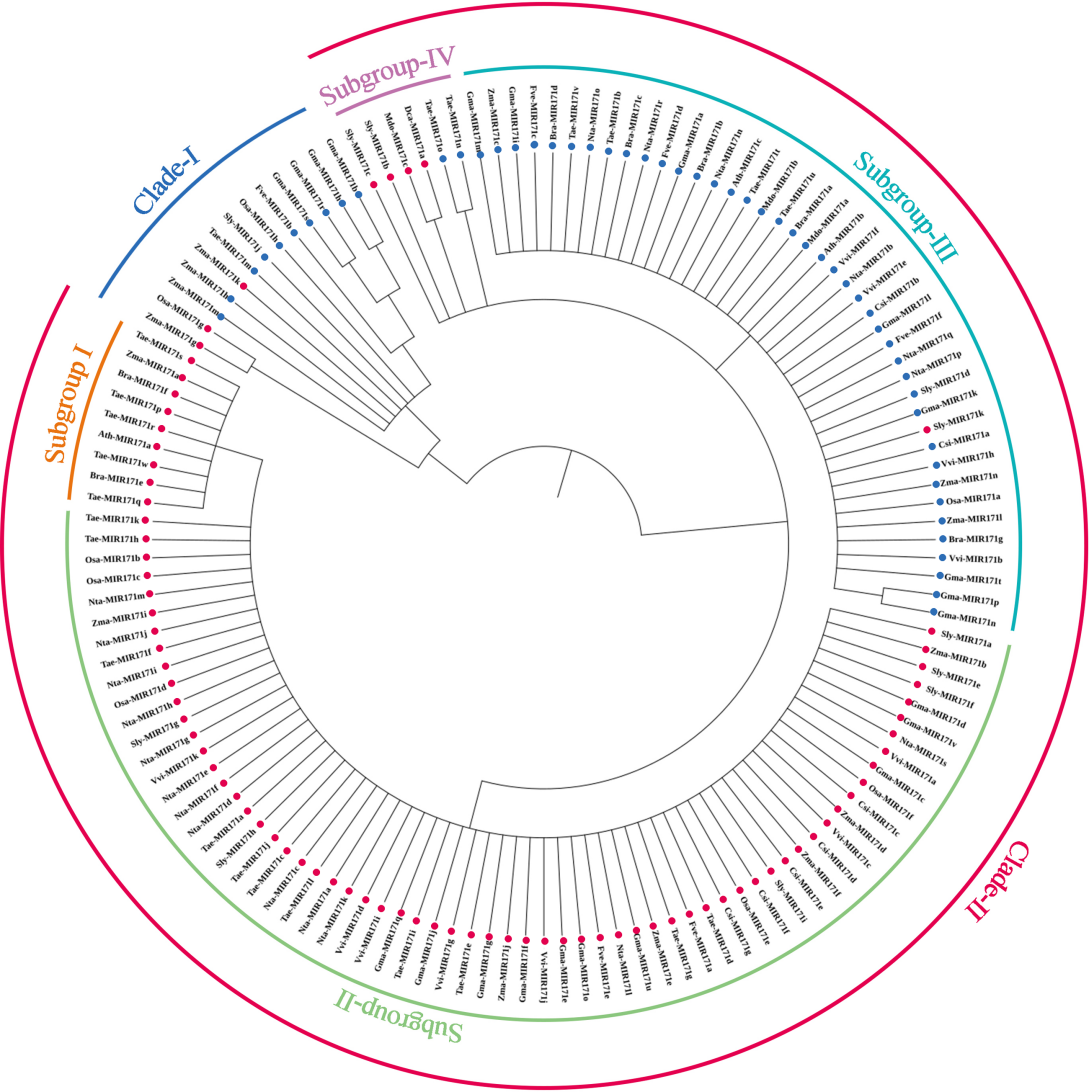
The maximum likelihood phylogenetic relationships among miR171 family members in diûerent species. (A) Thaliana (Ath), rice (Osa), strawberry (Fve), tomato (Sly), maize (Zma), wheat (Tae), cabbage (Bra), citrus (Csi), radish (Dca), soybean (Gma), apple (Mdo), grape (Vvi) and tobacco (Nta). Dots indicate where the corresponding precursors are Clades.

**Figure 3:**
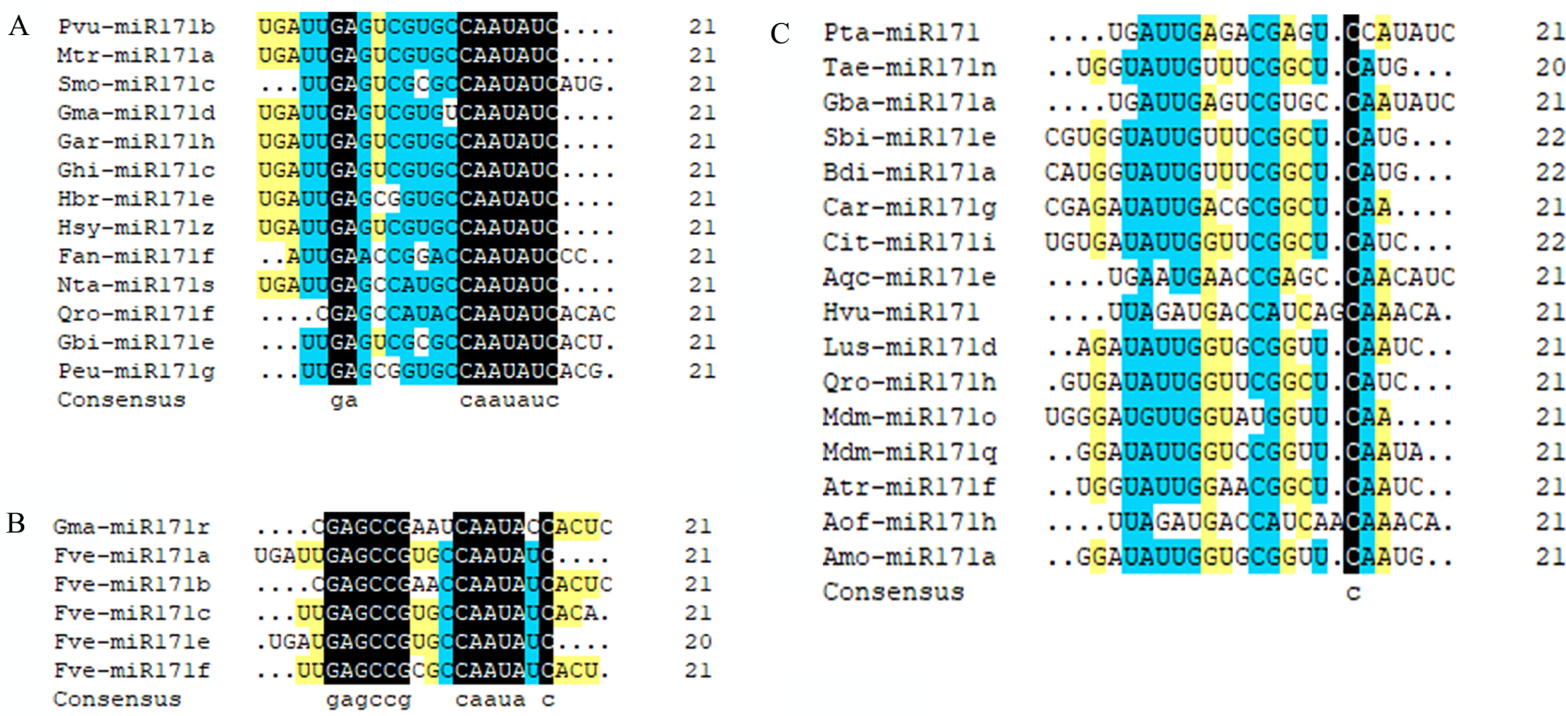
Multi sequence alignment of specially mature miR171. (A) Multiple sequence alignment of the mature miR171s without “GAGCCG”; (B) Multiple sequence alignment of the mature miR171s without “CAAUAU”; (C) Multiple sequence alignment of the mature miR171s without “GAGCCG” and “CAAUAU”. Black indicates a homology level of 100%, yellow indicates a homology level above 75%, and blue indicates a homology level above 50%.

We observed that mature sequences of miR171 in the same species have high sequence similarity and that some identical sequences exist between different species by sequencing all miR171 family members (Fig. 1B). Further, to gain an insight into the evolutionary process of the *miR171* family, several representative species were selected (*Arabidopsis thaliana*, *Oryza sativa*, *Zea mays*, *Triticum aestivum*, *Nicotiana tabacum*, *Solanum lycopersicum*, *Fragaria vesca*, *Vitis vinifera*, *Malus domestica*, *Glycine max*, *Citrus sinensis*, *Brassica rapa*, and *Daucus carota*) to construct the ML phylogenetic tree of *miR171* family members (Fig. 2). The ML phylogenetic tree obtained grouped *miR171* into two clades. Clade-I comprised 13 mature miRNAs, while the remaining 122 formed Clade-II. In Clade-II, the sequences of the same subgroups varied only by 4–6 bases, whereas those in Clade-I were identical. The low degree of sequence similarity within Clade-I implies higher sequence variation, and the low degree of sequence divergence within and between the two subgroups of Clade-II suggests that they have undergone consistent or similar evolutionary processes. The presence of *miR171* sequences from different species in each clade and subgroup further supports this observation. We also noted the occurrence of “GAGCCG” and “CAAUAU” segments in all clades. Even without feature fragments, there was some sequence similarity between the sequences (Fig. 3), which confirms the high conservation of *miR171* across different species.

### Target genes of miR171

MiRNAs function by binding to complementary sequences on target genes, which regulate gene expression through cleavage or translational repression. Various methods, such as degradome sequencing, 5’RACE, and site prediction, have been used to identify miR171 target genes. It has been discovered that most of the target genes of miR171 belong to the GRAS family through inductive summation. The GRAS gene family is exclusive to plants and comprises transcription factors containing a C-terminal GRAS domain (Waseem et al., 2022). The GRAS family has diverse functions and is widely distributed across plants. In addition to its role in regulating growth and development, its subfamilies are also involved in crucial processes such as signal transduction, stress response, and plant-microbe interaction (Silverstone, Ciampaglio & Sun, 1998; Schulze et al., 2010; Zhou et al., 2018a; Zhou et al., 2018b; Zhang et al., 2020; Gao, Wan & Wong, 2013).

The *GRAS* family member *LOST MERISTEMS 1 (LOM1/2/3)*, also known as *HAM1/2/3* or *SCL6-II/III/IV* in different species, regulates shoot apex indeterminacy and influences shoot branching (Engstrom et al., 2011; Llave et al., 2002; Xue et al., 2014; Sunkar & Zhu, 2004; Geng et al., 2021; Zhang et al., 2010; Stuurman, Jäggi & Kuhlemeier, 2002; Wang et al., 2010). When *LOM1* is combined with *ath-miR171c* and cleaved, resulting in its reduced expression, the plant exhibits a reduced branching phenotype (Wang et al., 2010). *Unigene83401*, a predicted target gene of *Pde-miR171*, was discovered to have a cleavage site and exhibited significant homology with *AtSCL6-III*. In addition, *Pde-miR171* can also direct target *AtSCL-III* and *AtSCL-* IV; to regulate leaf shape and root length (Hai et al., 2018; Wang et al., 2010). In addition, *SCL6* has been identified as a target gene of *miR171* in several plant species, including *Pinus densata*, *Larix kaempferi*, *Lilium pumilum*, *Hordeum vulgare*, *Oryza sativa*, *Glycine max*, *Pyrus communis*, and *Brassica oleracea* (Hai et al., 2018; Li et al., 2014; Yan et al., 2022; Curaba et al., 2013; Um et al., 2022; Hossain et al., 2019; Jiang et al., 2018b; Li et al., 2018).

Degradome sequencing has identified three *SCLs (CsSCL1/2/3*) as miR171c target genes in *Citrus callus* (Wu et al., 2015), and overexpression of *miR171c* in citrus reduced the expression of these genes and promoted somatic embryogenesis (Shi et al., 2022). Other targets of *miR171* include *PylSCL22*, *BolSCL27*, *SlGRAS24*, *MnoLOM2*, *MnoLOM3*, *GmNSP2*, *MtNSP2*, *LjaNSP2,* and *lncRNA* (Jiang et al., 2018a; Li et al., 2018; Huang et al., 2016; Sun et al., 2022; Hossain et al., 2019; Lauressergues et al., 2012; Luis et al., 2012; Yang et al., 2022).

miRNA/miRNA* is a duplex product after Dicer processing the *pre-miRNA* (Chen, 2005). The miRNA is named the guide strand and the miRNA* is named the passenger strand. During RNA-induced silencing complex (RISC) maturation, the miRNA/miRNA* duplex associates with the catalytic subunit, an ARGONAUTE (AGO) protein. The miRNA in the duplex directs gene silencing and is retained and incorporated into RISC, while the passenger strand degrades due to its low stability (Chen, 2005; Zuo et al., 2011). However, studies have shown the presence of active AGO1-*miR171a* * complexes. Although there are five mismatches between miR171* and *SUVH8*, *SUVH8* is identified as the target gene of miR171* in *Arabidopsis thaliana* using poly (A+) RNA from pistils and 5’RACE analysis (Manavella et al., 2013).

In addition, many other target genes have been predicted but not confirmed. We used the psRNATarget prediction website (http://www.zhaolab.org/psRNATarget/home) to analyze potential target genes for *miR171* in three representative plants: *Arabidopsis*, tobacco, and tomato. It was found that *miR171* can regulate pre-mRNA splicing factor-like proteins, programmed cell death 4, ABC transporter type 1, kinase, and other coding genes in addition to the above target genes (Table S1). These results provide new information about miR171 target genes and their corresponding functions.

## Functions of miR171

### Plant growth and development

#### Embryogenesis

Somatic embryogenesis (SE) is an important method for *in vitro* regeneration of plants (Shi et al., 2022). In recent years, many miRNAs involved in the SE systems of plants, including *Arabidopsis*, rice, and maize, were identified (Szyrajew et al., 2017; Luo et al., 2006; Chávez-Hernández et al., 2015). Shi et al. (2022) predicted that *csi-miR171* might play an important role in the embryogenic callus (EC) and during SE of citrus because of its abundant expression only when embryogenic callus was induced (Wu et al., 2011; Shi et al., 2022). At relatively low expression levels of the target gene SCLs, transgenic citrus plants overexpressing *csi-miR171c* produced massive somatic embryos earlier than wild-type plants. The number was proportional to the expression level (Shi et al., 2022). In larch, *miR171* was highly expressed in embryogenic cultures, but its expression was nearly undetectable in non-embryogenic cultures, whereas its target gene *LaSCL6* showed an opposite pattern (Li et al., 2014). Another study discovered that more *LaSCL6* transcripts were cleaved in EC, indicating that *miR171-SCL6* may have a post-transcriptional role in maintaining embryogenic potential (Zhang et al., 2010). The mismatch between *Lka-miR171a-e* and *SCL6* ranged from 0.5 to 2.5 (Li et al., 2014). Compared to the other members of the Larch miR171 family, *Lka-miR171d* has the highest frequency, indicating that it may be more critical for maintaining embryogenic potential (Zang et al., 2021).

Different members of the *miR171* family show differential expression in the two lily species at different EC stages, suggesting their complex regulation of *SCL6* (Li et al., 2017). Silencing *Lpu-miR171a/b* and overexpressing *LpSCL6-II/I* speed up the development of *Lilium* somatic embryos (Yan et al., 2022). It is worth mentioning that, unlike other miRNAs, *Lda-miR171a/c* and their target genes have a positive correlation (Li et al., 2017). *MiR171* expression is downregulated in radish and rice during embryogenesis, while it is predominantly expressed in EC before initiating rice plant differentiation (Zhai et al., 2014; Luo et al., 2006). In the EC of Japanese Larch, the expression of *Ptc-miR171c/d/j/k* is significantly increased compared to the non-embryogenic callus (Zhang et al., 2010). These conclusions suggest that *miR171* is essential for the development of plant regeneration, but its specific regulatory mechanism needs further investigation.

#### Vegetative development

Many studies have demonstrated that *miR171* plays a diverse and indispensable role in vegetative development. The *Hairy Meristem (HAM)* transcription factors, members of the GRAS family, have been identified as *miR171* targets and have been shown to significantly regulate shoot apical meristem and axillary meristem formation. *AtHAM* interacts with *WUS/WOX*, which plays a vital role in stem cell differentiation and maintenance in all meristem cells. *HAM* and *WUS* regulate common target genes that promote sprout stem cell proliferation. *HAM* and *WOX* are widely distributed throughout the plant, where their functions overlap, and they work together as regulators in different stem cell niches (Zhou et al., 2015). The combination of *WUS* and the *CLV3* promoter activates the expression of *CLV3*, forming a negative feedback regulatory loop that ensures the normal maintenance and transformation of *Arabidopsis thaliana*, but this activation is only effective in the absence of *HAM*. Ectopic miR171 expression results in anomalous SAMs and dysregulated CLV3 expression (Brand et al., 2000; Schulze et al., 2010; Zhou et al., 2018a; Zhou et al., 2018b). Besides its role in *Arabidopsis* meristem, HAM has also been reported to regulate meristem in *Petunia hybrida*, *Dendrocalamus latiflorus*, *Solanum lycopersicum*, and other plants (Stuurman, Jäggi & Kuhlemeier, 2002; Yang et al., 2014; Hendelman et al., 2016), as a target gene for miR171.

Furthermore, the epidermis-specific transcription factors *ATML1* and *PDF2* directly bind to the L1 box of the *miR171* promoter, forming a *HAM* concentration gradient, which regulates shoot development (Han et al., 2020). Overexpression of *Hvu-miR171a* in barley strongly represses the function of the target gene *HvSCL*, resulting in indeterminate initiation of the axillary meristem (Curaba et al., 2013). Similarly, increased expression of the target gene *SlGRAS24* leads to an abnormal axillary bud phenotype (Huang et al., 2016). *PpGRAS12*, the *miR171* target in *Physcomitrium patens*, leads to the formation of multiple apical meristems in the vegetative stage of the gametophyte when its transcript level increases (Beheshti et al., 2021). Thus, the miR171-HAM pathway plays an essential role in regulating the formation of apical and axillary meristems in plant shoots.

Overexpression of *miR171c* in *Arabidopsis* inhibits shoot branching, root elongation, and trichome distribution while promoting plant height, chlorophyll accumulation, and altered leaf shape and patterning, consistent with the target genes in the *scl6* triple mutant plants (Wang et al., 2010; Xue et al., 2014). Further research revealed that the target genes of the *miR171a* gene activate its expression, forming a feedback regulatory loop (Xue et al., 2014). Transgenic plants overexpressing *Ath-miR171a* and down-regulating *Sly-miR171* showed the opposite phenotype with increased branching (Song, Axtell & Fedoroff, 2010; Kravchik et al., 2019). Transgenic *Arabidopsis* also showed defects in cauline and rosette leaf patterns, and transgenic tomatoes produced irregular compound leaves (Song, Axtell & Fedoroff, 2010; Kravchik et al., 2019). Overexpression of *Hvu-miR171a* in barley resulted in shorter plants, fewer tillers, and more leaves due to repression *HvSCL* (Curaba et al., 2013).

The diameter of stems and the number of nodes increased significantly upon overexpression of Osa-miR171c mutants, while the leaf blades became irregular compared to the wild-type plants (Fan et al., 2015). In sugarcane, the length of internode increased along with the increase of *miR171* expression (Sternes & Moyle, 2014). Transgenic tomatoes with increased expression of *SlGRAS24*, the target of *Sly-miR171*, have shorter and narrower leaves without serrated edges and more branches. Additionally, transgenic plants showed significantly suppressed root length compared to wild-type plants (Huang et al., 2016). Excessive accumulation of *miR171* in *Medicago truncatula* promotes primary root development and reduces lateral root growth by targeting *NSP2* (Lauressergues et al., 2015).

In contrast to its target *SCL6*, the expression of *miR171* in *Arabidopsis* increased significantly during the light period while decreasing quickly during the dark period (Siré et al., 2009). However, the circadian clock does not regulate the accumulation of *miR171* (Siré et al., 2009). Many cis-acting regulatory elements for photosensitivity are involved in the 2000 bp promoter sequences of Ptc-*miR171*, suggesting that its members may participate in light signal transduction and light morphogenesis in diverse ways (Liu et al., 2014).

#### Phase transition

*MiR171* is also involved in the regulation of plant phase transitions. Phase transitions in plants are crucial stages in their growth and development and are closely related to plant resistance to disease and insects, yield, quality, and other agronomic traits. This process is generally non-directional and irreversible (Hu et al., 2022). Therefore, identifying the variables in this process is necessary.

Transgenic barley overexpressing *Hvu-miR171a* showed delayed phase transitions under long-day conditions, with more leaves, shorter internode lengths, and later flowering compared to wild-type plants. These phenotypic changes are partly due to the upregulation of *miR156* in transgenic plants (Curaba et al., 2013). The mutant of rice, which up-regulated the expression of the *Osa-miR171c*, continued to produce new leaves. At the same time, the wild-type plant converted into an inflorescence meristem, indicating the phase transition’s delayed was because of the changes of *Osa-miE171c* and its target gene *OsHAM* expression (Fan et al., 2015). Overexpression of *Sly-miR171* in tomatoes accelerates the phase transition and plant height (Huang et al., 2016). Fluctuations in *miR171* levels during phase transition may be an effective pathway for yield improvement, and it also provides new ideas for plant breeding.

#### Reproduction development

Several studies have demonstrated that miRNAs are involved in reproductive development. Overexpression of *miR171* in barley results in late flowering and sterile spikes due to delayed differentiation of spikelet meristems into floral organs (Curaba et al., 2013). Overexpression of 35Spro-miR171c in Arabidopsis resulted in the formation of abnormal and late flowers under long-day conditions (Wang et al., 2010). In rice, upregulation of *Osa-miR171c* significantly delayed flowering time, caused pleiotropic morphological abnormalities in the panicle, and affected SAM maintenance (Fan et al., 2015). When *SlGRAS24*, the target gene of *Sly-miR171*, was up-regulated (OE-SlGRAS24), flower opening was late, and the fruits were smaller with fewer seeds due to damaged pollen sacs and fewer viable pollen grains (Huang et al., 2016). Consistent with the above observation, the transgenic tomato with down-regulated *Sly-miR171* showed male sterility because of malformed and nonviable pollen (Kravchik et al., 2019). Overexpression of *miR171* and down-regulation of its target gene, *SCL*, also contributes to the reproductive development of rice. *MiR171* up-regulated transgenic rice plants had thicker tillers, longer panicles, and more spikelets than wild-type plants (Tong et al., 2017).

#### Phytohormone response

According to several studies, *miR171* also plays a role in the phytohormone response of plants. In *Arabidopsis*, the *miR171* target gene *SCL2* 7 can reverse-regulate *miR171* and create a feedback loop essential for mediating gibberellin signaling and regulating chlorophyll biosynthesis and leaf growth (Ma et al., 2014). Furthermore, the 1,000-bp upstream promoter sequence of *miR171* was analyzed, which revealed the presence of several cis- and trans-acting elements for phytohormone response, including TGA-element for auxin, GARE motif for gibberellin, ERE for ethylene, and TCA-element for salicylic acid (Liu et al., 2008). Likewise, the 2000-bp upstream promoter sequence of Pts-miR171 also contains cis-acting elements for salicylic acid (TCA-element) and abscisic acid (ABRE) responsiveness (Liu et al., 2014). In pear, IAA-induced *miR171* negatively regulates the IAA signaling cascade by targeting *PyrSCL6/22*; thus, promoting shoot growth and maintaining apical dominance (Jiang et al., 2018b). *SlGRAS24*, the target gene of *Sly-miR171*, also responds to auxin, suggesting that *Sly-miR171* is involved in tomato growth and development *via* the auxin-signaling pathway (Huang et al., 2016).

**Figure 4:**
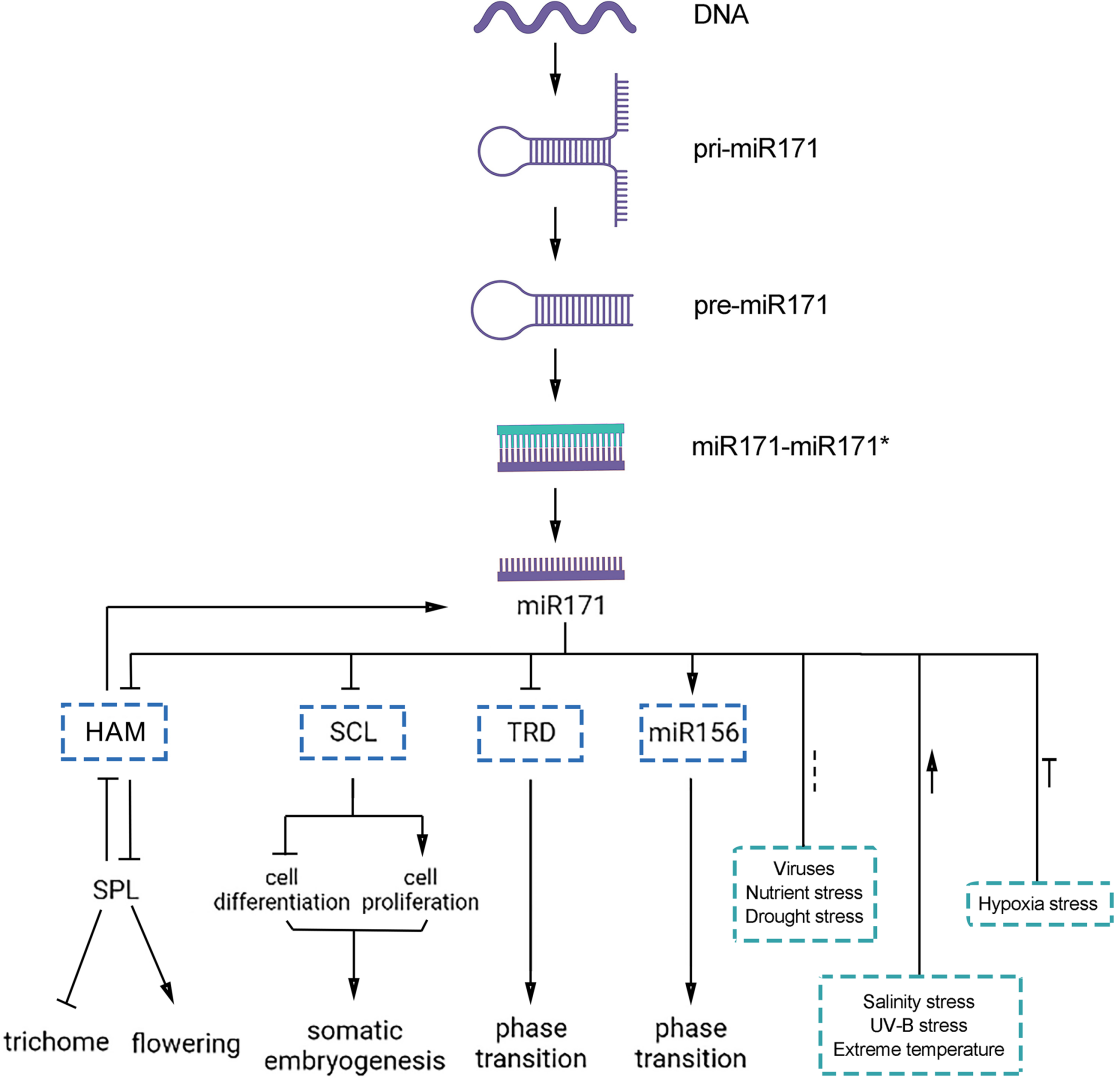
Functions of *miR171* and its target genes in plants. Arrows indicate upregulation, blunted lines indicate downregulation, and dotted lines indicate undefined correlations or functions.

Overall, *miR171* is critical for all stages of plant growth and development. *MiR171* and its target genes play essential regulatory roles throughout the life cycle of a plant, from embryonic development to vegetative growth to reproductive growth (Table 1, Fig. 4). Therefore, understanding the mechanisms by which *miR171* and its target genes influence plant growth and development is crucial for crop breeding and enhancing crop yield and quality.

**Table 1:** Recent updates on growth development related functions of miR171.

Species	Biological processes and the expression changes of miR171 (↑up, ↓ down)	Target gene	References
*Arabidopsis thaliana*	Root and leaf development, meristem formation, trichome distribution, diurnal cycle, chlorophyll biosynthesis, flower development, phytohormone	SCL6-II/III/IV SUVH8 SCL27	Wang et al. (2010), Xue et al. (2014), Han & Zhou (2022), Manavella et al. (2013), Ma et al. (2014), Siré et al. (2009), Liu et al. (2008)
*Oryza sativa*	Somatic embryogenesis, phase transition, root development, chlorophyll accumulation	SCL	Luo et al. (2006), Fan et al. (2015), Cho & Paszkowsk (2017), Tong et al. (2017)
*Citrus*	Somatic embryogenesis	SCL	Shi et al. (2022)
*Larix leptolepis*	Somatic embryogenesis	SCL	Zhang et al. (2010)
*Lilium*	Somatic embryogenesis	*SCL6*	Li et al. (2017), Yan et al. (2022)
*Raphanus sativus*	Somatic embryogenesis	*SCL6*	Zhai et al. (2014)
*Larix kaempferi*	Somatic embryogenesis	*SCL6*	Zang et al. (2021)
*Solanum lycopersicum*	Anther development, shoot branching, leaf architecture, plant height, flowering time, root development, fruit set and development, phytohormone, phase transition	*HAM,* *NSP2L,* *GRAS24*	Kravchik et al. (2019), Huang et al. (2016), Hou et al. (2019)
*Hordeum vulgare*	Plant height, leaf numbers, phase transition, floral meristem determinancy	*SCL*	Curaba et al. (2013)
*Saccharum officinarum*	Stem	*SCL6*	Sternes & Moyle (2014)
*Physcomitrium patens*	Meristem formation	*GRAS12*	Beheshti et al. (2021)
*Medicago truncatula*	Root development	*NSP2*	Lauressergues et al. (2015), Branscheid et al. (2011)
*Populus trichocarpa*	phytohormone	*GRAS* *MYB*	Liu et al. (2014)
*Pyrus*	Phytohormone shoot growth	*SCL6/22*	Jiang et al. (2018b)
*Pinus densata*	Needle, stem, root, leaf, flower	*SCL6*	Hai et al. (2018)
*Dendrobium officinale*		*SCL*	Yang et al. (2015)
*Litchi chinensis*		*GRAS8/9/24/27*	Chen et al. (2021)

### Environmental stress

#### Abiotic stress response

A plant will inevitably experience a variety of environmental stresses throughout its life cycle, such as drought, excessive heavy metal exposure, nutrient deficiency, etc., which significantly affect plant growth and development. Microarray data identified 14 stress-inducible *miRNAs*, including *miR171*, in *Arabidopsis* under drought, low temperature, and high salinity conditions (Liu et al., 2008). However, after eight hours of drought stress, wheat roots displayed an opposite trend with the down-regulation of *miR171* (Liu et al., 2008; Kantar, Lucas & Budak, 2011; Sunkar & Zhu, 2004). The expression pattern of *miR171* in rice during drought stress is stage- dependent; the expression is higher during the tillering stage and lower during the reproductive stage (Zhou et al., 2010). While *Osa-pre-miR171a* abundance decreases under drought stress, *Osa-miR171f* expression levels rise, and it cleaves SCL6-I/II transcripts to alleviate drought symptoms (Chung et al., 2016; Um et al., 2022). The expression of *Osa-miR171i* decreases after ten days of water withholding to cope with water scarcity, as evidenced by Zhou et al. (2010). Although *miR171* expression levels in tobacco cultivars vary, no clear pattern of change is observed under drought stress (Deniz, 2015). Drought stress leads to reduced *miR171* expression in *Triticum dicoccoides*, *Medicago truncatula*, *Populus tomentosa*, and *Ipomoea campanulata*, increased expression in *Morus alba*, and a mixed pattern of expression in *Solanum tuberosum* and *Prunus persica*, as reported by Kantar, Lucas & Budak (2011), Wang et al. (2011), Ren et al. (2012), Ghorecha et al. (2017), Sun et al. (2022), Hwang et al. (2011), Eldem et al. (2012), and Ferdous, Hussain & Shi (2015). These findings suggest that *miR171* plays an essential role in regulating multiple signaling pathways related to drought response.

Tomatoes exhibit leaf chlorosis, chlorophyll and starch degradation, and a decline in photosynthetic efficiency when exposed to carbon starvation; however, the symptoms alleviate when melatonin-induced *miR171b* is overexpressed and targets the *GWD* gene (Wang et al., 2022). Zinc oxide nanoparticles (ZnONPs) are the most widely used nanomaterials that can improve plant resistance to drought, cadmium, arsenic, etc. (Niazi et al., 2022; Semida et al., 2021; Hussain et al., 2018; Wu et al., 2020). The down-regulation of miR171 was observed in wheat when treated with 10 mg/L ZnONPs, whereas the up-regulation of *miR171* was observed when the dosage was increased to 50 mg/L (Niazi et al., 2022). The expression of *miR171* in leaf tissues of *Arabidopsis thaliana* was up-regulated with a rise in temperature from 35 °C to 45 °C (Mahale et al., 2014), suggesting that temperature is also a factor that affects miR171 expression levels. *Ath-miR171* is also induced upon nitrogen starvation and suppresses *SCL6-II/III/IV* in response to better development of the primary root system (Liang, He & Yu, 2012). Boron treatment reduces the number of root tips, which further affects the architecture of the root system. B-toxic treatment in citrus reduces the expression levels of *miR171* while increasing *SCL* expression to protect against stressful environments (Huang et al., 2019). Chromium stress initially decreased the expression of *miR171* in rice, but after 24 hours, it began to increase (Dubey et al., 2020). A study showed that wheat *miR171* reaches its maximum expression 2 h after UV-B treatment and interacts with other miRNAs in response to a stressful environment (Wang et al., 2013). Hypoxia treatment decreases the expression of *miR171* in tomato roots and increases the length and quantity of lateral roots (Hou et al., 2019). When GSNO was used to mimic environmental NO, an increase in the abundance of both mature and precursor *miR171* was observed (Santos et al., 2022). Since NO is essential for root hair development in *Arabidopsis*, it is plausible that *miR171* will encourage root hair development when GSNO is present (Moro et al., 2017; Santos et al., 2022).

Several investigations have explored the involvement of *miR171* and its target genes in the plant’s response to abiotic stress (as shown in Table 2 and Fig. 4). Nonetheless, some questions remain unanswered, such as how *miR171* collaborates with other miRNAs to regulate the same stress and whether there is any correlation among the various target genes controlled by *miR171*.

**Table 2:** Recent updates on stress response related functions of miR171.

Species	Biological processes and the expression changes of miR171 (↑up, ↓ down)	Target gene	References
*Arabidopsis thaliana*	Nitrogen starvation ↑, heat stress ↑, high salinity stress ↑, drought stress ↑, temperature stress ↑, GSNO stress ↑	*SCL6*	Liang, He & Yu (2012), Mahale et al. (2014), Liu et al. (2008), Santos et al. (2022)
*Oryza sativa*	Drought stress ↑↓, bacterial infection ↑, chromium stress ↓↑	*SCL;* *ACP1*	Zhou et al. (2010), Tong et al. (2017), Dubey et al. (2020), Sasani et al. (2020)
*Solanum lycopersicum*	Hypoxia stress ↓, arbuscular mycorrhizal symbiosis ↑	*GRAS24*	Hou et al. (2019), Wu et al. (2016)
*Populus tomentosa*	Drought stress ↓, flooding stress ↓		Ren et al. (2012)
*Medicago truncatula*	Drought stress ↓, arbuscular mycorrhizal symbiosis ↑	*GRAS;* *NSP2*	Wang et al. (2011), Lauressergues et al. (2012)
*Prunus persica*	Drought stress ↑		Eldem et al. (2012)
*Solanum tuberosum*	Drought stress ↓↑	*GRAS*	Hwang et al. (2011)
*Spartina alterniflora*	Salinity stress ↓		Qin et al. (2015)
*Ipomoea campanulata*	Drought stress ↓		Ghorecha et al. (2017)
*Morus alba*	Salinity stress ↑, drought stress ↑	*LOM2; LOM3*	Sun et al. (2022)
*Citrus*	Boron toxicity ↓, bacterial infection ↓	*SCL*	Huang et al. (2019), Reyes et al. (2015)
*Triticum aestivum*	UV-B stress ↑, Zinc oxide nanoparticles ↓↑	*GRAS*	Wang et al. (2013), Niazi et al. (2022)
*Nitotiana tabacum*	Drought stress ↓	*SCL*	Adalı (2015)
*Glycine max*	Bacterial infection ↑↓		Hossain et al. (2019)
*Hibiscus cannabinus*	Bacterial infection	*SCL1*	Gao, Wan & Wong (2013)
*Lotus japonicus*	Arbuscular mycorrhizal symbiosis ↑	*NSP2*	Luis et al. (2012)

#### Plant-microbe interactions

Studies on viral infection and symbiosis have revealed a complex reciprocal relationship between plants and microbes, and miRNA plays a crucial role in these processes. For instance, interactions between soybean and *Bradyrhizobium japonicum* led to the overexpression of *miR482* and *miR1515*, causing an increase in the number of nodulations in soybean (Li et al., 2010). Several other reports have investigated the role of *mir171* in plant-microbe interactions. In response to infection with the bacterium *B. japonicum*, *Gma-miR171o,* and *Gma-miR171q* display opposite expression patterns; *Gma-miR171q* shows up-regulation while *Gma-miR171o* shows down-regulation (Hossain et al., 2019). *Rhizoctonia solani* infection, which simulates sheath blight disease in rice, leads to an increase in O*sa-miR171* expression in the later stages of stress (Sasani et al., 2020).

On the other hand, infection with the rice stripe virus results in down-regulation of *Osa-miR171b*. At the same time, overexpression of this miRNA confers resistance to the virus and mitigates virus-induced symptoms (Tong et al., 2017). Compared to control plants, Hibiscus chlorotic ringspot virus infection of kenaf up-regulates *miR171*, while the corresponding target gene *SCL1* shows opposite changes in expression (Gao, Wan & Wong, 2013). The *miR171* in sweet orange shows almost 3-fold lower expression in plants infected with *Citrus psorosis virus* and a corresponding enrichment of the target gene *SCL6* (Reyes et al., 2015). This change led to the relative accumulation of the target gene *SCL6* (Reyes et al., 2015). Further immunoprecipitation experiments showed the interaction between *pre-miR171a* and viral protein (Reyes et al., 2015).

Previous research has also investigated the function of *miR171* in regulating the symbiotic relationship between plants and microbes. For instance, in *Lotus japonicas*, the expression of *Lja-miR171c* is higher in infected nodules than in healthy ones, indicating its potential involvement in bacterial infection (Luis et al., 2012). In *Medicago truncatula*, overexpression of *miR171* h results in a nearly 50% decrease in arbuscular mycorrhizal colonization, similar to that observed in the *nsp2* mutant (Lauressergues et al., 2012). In addition, *miR171* h-NSP2 is crucial for the Myc-LCO signaling pathway (Lauressergues et al., 2012). Interestingly, *miR171b* enhances mycorrhization by promoting the expression of the target gene *LOM1* and shielding it from cleavage by other miR171 family members (Couzigou et al., 2016). Tomato *miR171 g*, which shares a high sequence similarity with *Mtr-miR171 h*, was also up-regulated upon *Rhizophagus irregularis* inoculation treatment. Moreover, the same trend was observed for *miR171i*, suggesting a role in regulating arbuscular mycorrhizal symbiosis (Wu et al., 2016). Thus, different miR171 members play different roles in the same biological process, controlling plant-microbe symbiosis through many targets.

#### Interaction between the miR171 network and other small-RNAs

Several studies have shown the complex interactions between the *miR171* regulatory network and other miRNAs. Transgenic maize with overexpressing *miR171* and *miR156* has a similar phenotype of prolonged juvenile development (Chuck et al., 2007a; Chuck et al., 2007b). Since *miR156b/c* overexpression in maize down-regulates *miR172*, *miR171* may also affect the miR156-miR172 pathway (Chuck et al., 2007b; Chuck, Meeley & Hake, 2008). Barley also showed similar results. Transgenic plants with up-regulation of *miR171* (OE171) showed a similar phenotype as transgenic plants with down-regulation of *miR172* (Curaba et al., 2013; Brown & Bregitzer, 2011). Moreover, in the inflorescence tissue of OE171, the expression of *miR156* increases but not that of *miR172*, whereas the target gene of *miR156, SCL* is also down-regulated (Curaba et al., 2013). Interestingly, in young leaves of OE171, increased *miR156* accumulation was accompanied by decreased *miR172* expression, suggesting that *miR171* affects the phase transition through the regulation of *miR156* and *miR172* in a spatiotemporally dependent manner (Curaba et al., 2013). In *Arabidopsis*, overexpression of *miR171* significantly reduced trichomes on the stem, and its target genes, *LOMs*, had an antagonistic effect on the *SPLs*, the target genes of *miR156* (Xue et al., 2014). It altogether suggests an indirect interaction between *miR171* and *miR156*. The stem cell niche (SCN) is composed of the quiescent center (QC), also known as the root stem cell niche, and the surrounding initial stem cells (Pardal & Heidstra, 2021). SCARECROW(*SCR*) is involved in the regulation of the SCN and plays a role in the specification and maintenance of the QC. In addition, *miR396* also plays a role in maintaining the SCN (Pardal & Heidstra, 2021). Based on these studies, we hypothesize that some degree of interaction exists between *miR171* and *miR396* for root system development.

The construction of a ceRNA network associated with drought resistance in rice identified a lncRNA *(MSTRG.28732.3)* that functions as a target gene for *miR171*. According to RT-qPCR analysis, the expression patterns of both genes were found to be inversely correlated (Yang et al., 2022). In response to salt stress, the screening of differentially expressed lncRNAs using ssRNA sequencing on duckweed with and without salt treatment revealed that some lncRNAs might act as target genes that interact with *miR171* to provide protection against salt stress (Fu et al., 2020). Additionally, studies indicate that specific lncRNAs are also targeted by multiple miRNAs; for instance, *TCNOS_00033722* is targeted by *miR156*, *miR169*, and *miR393*, while *TCONS_00044328* and *TCONS_00059333* are targeted by *miR171*, *miR167*, and *miR168* (Fu et al., 2020). Interestingly, the lncRNA *TCONS_00155383* acts as a precursor of *miR171* in polyploid switchgrass (Yan et al., 2018). Mutations in *SCR*, a predicted target gene for *miR171*, result in proliferation and abnormal differentiation of the bundle sheath cells, which suggests that both *miR171* and lncRNA are involved in stem cell development (Yan et al., 2018; Slewinski et al., 2012).

## Conclusions

*MiR171*, one of the earliest miRNAs discovered in plants, has a length of 20 to 23 nts and is one of the oldest and most evolutionarily conserved miRNAs. The fragments “GAGCCG” and “CAAUAU” are mostly conserved across all *miR171* sequences. *MiR171* plays a role in plant growth and development, hormone signaling, stress responses, and microbial interactions (Xue et al., 2014; Huang et al., 2016; Sun et al., 2022; Sasani et al., 2020). Its target genes are present across different gene families; however, the most common targets belong to the GRAS family of transcription factors, along with a small number of miRNAs, lncRNAs, and other genes (Han & Zhou, 2022; Xue et al., 2014; Yang et al., 2022; Luis et al., 2012). The wide range of its target genes can also account for the functional diversity of *miR171*.

As shown in the 1st, 3rd, 4th, and 12th rows of Table 1, *miR171* can act on different target genes and play different roles in the same species. Moreover, *miR171* has been found to target the same or different genes to achieve similar effects across various species. In *Arabidopsis*, *miR171* cleaves *SCL6-II/III/IV* and *SCL27* to regulate chlorophyll biosynthesis and organ development (Xue et al., 2014; Ma et al., 2014). Even ath-miR171* has been observed to target *SUVH8*, thereby influencing plant height and leaf type (Manavella et al., 2013). In both *Citrus* and *Larix leptolepis*, miR171 targets SCL to regulate somatic embryogenesis (Shi et al., 2022; Zhang et al., 2010). In *Medicago truncatula*, *miR171* targets *NSP2* to regulate root development, but in *Arabidopsis*, *miR171* exerts the same function by regulating another target gene, *SCL6* (Lauressergues et al., 2015; Wang et al., 2010). In addition, different propagation patterns of the same species can also lead to differences in *miR171* levels. For example, micro-propagated *D. officinale* has much higher levels of *miR171* than conventional cultivars, which also accounts for the difference in their growth rates (Yang et al., 2015). In addition, miR171 and its target genes may exhibit varying patterns of accumulation in response to different levels of stress. For instance, the expression of miR171 is decreased at low concentrations of ZnONPs, while high concentrations lead to its upregulation, indicating the existence of a minimum threshold for *miR171* expression (Niazi et al., 2022). The same stress in different species also causes variation in expression levels of *miR171*. In Table 2, 1st, 4th to 7th, 10th, 11th and14th rows demonstrate that, in response to drought stress, *miR171* expression is down-regulated in *Triticum dicoccoides*, *Populus tomentosa*, *Medicago truncatula*, and up-regulated in *Arabidopsis*, *Prunus persica*, and *Morus alba* (Kantar, Lucas & Budak, 2011; Ren et al., 2012; Wang et al., 2011; Liang, He & Yu, 2012; Eldem et al., 2012; Sun et al., 2022).

Overall, this review summarizes the information on the role of *miR171* in plant development and its response to external negative stimuli. However, a clear picture of how different *miR171* members interact with their common targets in the same plant species while participating in biological processes is still lacking. In addition, the relevant regulatory mechanisms for the passenger strand and uplink-downlink networks are still unclear and need further exploration. Despite our limited understanding of the *miR171* association network and its interacting genes or proteins, the collective functions of *miR171*, such as somatic embryogenesis, phytohormone signaling responses, and plant-microbe interactions, are gradually becoming clear.

##  Supplemental Information

10.7717/peerj.15632/supp-1 Table S1 miR171 target gene prediction results

## Additional Information and Declarations

### Data Availability

The following information was supplied regarding data availability: The data is available at PmiREN: miR171
https://www.pmiren.com/browsehref1search?stype=1&kw=miR171.
