# Supplementary material for: Role of microRNA miR171 in plant development
Source: PeerJ. 2023 Jul 10;11:e15632. doi: 10.7717/peerj.15632 (PMC10340099; doi:10.7717/peerj.15632)
Supplement: Table S1 [file peerj-11-15632-s001.docx]

| MiR171 | Sequence | Expect | Target Acc | **Target Description** |
| --- | --- | --- | --- | --- |
| Ath-MIR171a | UGAUUGAGCCGCGCCAAUAUC | 0.0 | AT4G00150.1 | Transcription factor GRAS |
|  |  | 0.0 | AT3G60630.1 | Transcription factor GRAS |
|  |  | 0.0 | AT2G45160.1 | Transcription factor GRAS |
|  |  | 3.0 | AT5G62560.1 | RING/U-box superfamily protein with ARM repeat domain-containing protein |
|  |  | 3.0 | AT4G11270.2 | Transducin/WD40 repeat-like superfamily protein |
|  |  | 3.0 | AT4G11270.1 | Transducin/WD40 repeat-like superfamily protein |
| Ath-MIR171b | UUGAGCCGUGCCAAUAUCACG | 1.5 | AT4G00150.1 | Transcription factor GRAS |
|  |  | 1.5 | AT3G60630.1 | Transcription factor GRAS |
|  |  | 1.5 | AT2G45160.1 | Transcription factor GRAS |
|  |  | 1.5 | AT3G47170.1 | HXXXD-type acyl-transferase family protein |
|  |  | 3.0 | AT5G23250.1 | Succinyl-CoA ligase%2C alpha subunit |
|  |  | 3.0 | AT5G23250.2 | Succinyl-CoA ligase%2C alpha subunit |
|  |  | 3.0 | AT5G23250.3 | Succinyl-CoA ligase%2C alpha subunit |
|  |  | 3.0 | AT2G32800.1 | protein kinase family protein |
|  |  | 3.0 | AT5G65720.2 | nitrogen fixation S (NIFS)-like 1 |
|  |  | 3.0 | AT5G65720.1 | nitrogen fixation S (NIFS)-like 1 |
|  |  | 3.0 | AT5G65720.3 | nitrogen fixation S (NIFS)-like 1 |
| Nta-MIR171a | UGAUUGAGCCGUGCCAAUAUC | 0.5 | Nitab4.5_0000312g0120.1 | Transcription factor GRAS |
|  |  | 0.5 | Nitab4.5_0012398g0020.1 | Transcription factor GRAS |
|  |  | 0.5 | Nitab4.5_0012398g0010.1 | Transcription factor GRAS |
|  |  | 0.5 | Nitab4.5_0000312g0160.1 | Transcription factor GRAS |
|  |  | 0.5 | Nitab4.5_0000859g0260.1 | Transcription factor GRAS |
|  |  | 0.5 | Nitab4.5_0011278g0010.1 | Transcription factor GRAS |
|  |  | 1.0 | Nitab4.5_0001279g0120.1 | Transcription factor GRAS |
|  |  | 1.0 | Nitab4.5_0002128g0050.1 | Transcription factor GRAS |
|  |  | 2.0 | Nitab4.5_0000223g0070.1 | ABC transporter type 1 |
|  |  | 2.5 | Nitab4.5_0002564g0030.1 | ABC transporter type 1 |
| Nta-MIR171b | UUGAGCCGCGCCAAUAUCACU | 0.0 | Nitab4.5_0000312g0120.1 | Transcription factor GRAS |
|  |  | 0.0 | Nitab4.5_0012398g0020.1 | Transcription factor GRAS |
|  |  | 0.0 | Nitab4.5_0012398g0010.1 | Transcription factor GRAS |
|  |  | 0.0 | Nitab4.5_0000312g0160.1 | Transcription factor GRAS |
|  |  | 1.0 | Nitab4.5_0000859g0260.1 | Transcription factor GRAS |
|  |  | 1.0 | Nitab4.5_0011278g0010.1 | Transcription factor GRAS |
|  |  | 2.0 | Nitab4.5_0000193g0120.1 | Transcription factor GRAS |
|  |  | 2.5 | Nitab4.5_0009190g0010.1 | Transcription factor GRAS |
|  |  | 2.5 | Nitab4.5_0001279g0120.1 | Transcription factor GRAS |
|  |  | 2.5 | Nitab4.5_0002128g0050.1 | Transcription factor GRAS |
|  |  | 3.0 | Nitab4.5_0000207g0400.1 | Protein of unknown function DUF538 |
|  |  | 3.0 | Nitab4.5_0004582g0080.1 | Aluminum-activated malate transporter |
| Nta-MIR171n | UUGAGCCGUGCCAAUAUCACG | 0.5 | Nitab4.5_0000312g0120.1 | Transcription factor GRAS |
|  |  | 0.5 | Nitab4.5_0012398g0020.1 | Transcription factor GRAS |
|  |  | 0.5 | Nitab4.5_0012398g0010.1 | Transcription factor GRAS |
|  |  | 0.5 | Nitab4.5_0000312g0160.1 | Transcription factor GRAS |
|  |  | 1.0 | Nitab4.5_0001279g0120.1 | Transcription factor GRAS |
|  |  | 1.0 | Nitab4.5_0002128g0050.1 | Transcription factor GRAS |
|  |  | 1.5 | Nitab4.5_0011278g0010.1 | Transcription factor GRAS |
|  |  | 1.5 | Nitab4.5_0000859g0260.1 | Transcription factor GRAS |
|  |  | 2.0 | Nitab4.5_0000193g0120.1 | Transcription factor GRAS |
|  |  | 2.5 | Nitab4.5_0009190g0010.1 | Transcription factor GRAS |
|  |  | 3.0 | Nitab4.5_0000207g0400.1 | Protein of unknown function DUF538 |
|  |  | 3.0 | Nitab4.5_0010236g0010.1 | Protein of unknown function DUF538 |
|  |  | 3.0 | Nitab4.5_0002263g0010.1 | Remorin, C-terminal |
|  |  | 3.0 | Nitab4.5_0000604g0150.1 | Remorin, C-terminal |
|  |  | 3.0 | Nitab4.5_0002887g0090.1 | Serine-threonine/tyrosine-protein kinase catalytic domain |
| Nta-MIR171r | UUGAGCCGUGCCAAUAUCACU | 0.5 | Nitab4.5_0000312g0120.1 | Transcription factor GRAS |
|  |  | 0.5 | Nitab4.5_0012398g0020.1 | Transcription factor GRAS |
|  |  | 0.5 | Nitab4.5_0012398g0010.1 | Transcription factor GRAS |
|  |  | 0.5 | Nitab4.5_0000312g0160.1 | Transcription factor GRAS |
|  |  | 1.0 | Nitab4.5_0002128g0050.1 | Transcription factor GRAS |
|  |  | 1.0 | Nitab4.5_0002128g0050.1 | Transcription factor GRAS |
|  |  | 1.5 | Nitab4.5_0000859g0260.1 | Transcription factor GRAS |
|  |  | 1.5 | Nitab4.5_0011278g0010.1 | Transcription factor GRAS |
|  |  | 2.0 | Nitab4.5_0000193g0120.1 | Transcription factor GRAS |
|  |  | 2.5 | Nitab4.5_0009190g0010.1 | Transcription factor GRAS |
|  |  | 3.0 | Nitab4.5_0010236g0010.1 | Protein of unknown function DUF538 |
|  |  | 3.0 | Nitab4.5_0000207g0400.1 | Protein of unknown function DUF538 |
|  |  | 3.0 | Nitab4.5_0002263g0010.1 | Remorin, C-terminal |
|  |  | 3.0 | Nitab4.5_0000604g0150.1 | Remorin, C-terminal |
|  |  | 3.0 | Nitab4.5_0002887g0090.1 | Serine-threonine/tyrosine-protein kinase catalytic domain |
| Nta-MIR171s | UGAUUGAGCCAUGCCAAUAUC | 2.0 | Nitab4.5_0000312g0120.1 | Transcription factor GRAS |
|  |  | 2.0 | Nitab4.5_0012398g0020.1 | Transcription factor GRAS |
|  |  | 2.0 | Nitab4.5_0012398g0010.1 | Transcription factor GRAS |
|  |  | 2.0 | Nitab4.5_0000312g0160.1 | Transcription factor GRAS |
|  |  | 2.0 | Nitab4.5_0000859g0260.1 | Transcription factor GRAS |
|  |  | 2.0 | Nitab4.5_0011278g0010.1 | Transcription factor GRAS |
|  |  | 2.5 | Nitab4.5_0001279g0120.1 | Transcription factor GRAS |
|  |  | 2.5 | Nitab4.5_0002128g0050.1 | Transcription factor GRAS |
|  |  | 1.5 | Nitab4.5_0000223g0070.1 | ABC transporter type 1 |
|  |  | 2.0 | Nitab4.5_0002564g0030.1 | ABC transporter type 1 |
|  |  | 3.0 | Nitab4.5_0001558g0040.1 | Oligopeptide transporter |
|  |  | 3.0 | Nitab4.5_0000309g0320.1 | Tetratricopeptide repeat |
|  |  | 3.0 | Nitab4.5_0010615g0020.1 | Protein kinase-like domain |
|  |  | 3.0 | Nitab4.5_0002551g0010.1 | Protein kinase-like domain |
|  |  | 3.0 | Nitab4.5_0002315g0150.1 | Translation elongation factor |
|  |  | 3.0 | Nitab4.5_0007587g0020.1 | Peptidase S8 |
|  |  | 3.0 | Nitab4.5_0008253g0010.1 | Major facilitator superfamily domain |
|  |  | 3.0 | Nitab4.5_0006241g0040.1 | Bulb-type lectin domain |
| Sly-MIR171a | UGAUUGAGCCGUGCCAAUAUC | 0.5 | Solyc08g078800.1.1 | Transcription factor GRAS |
|  |  | 0.5 | Solyc01g090950.3.1 | Transcription factor GRAS |
|  |  | 2.5 | Solyc02g085600.1.1 | ABC transporter family protein |
|  |  | 2.5 | Solyc08g081890.3.1 | ABC transporter family protein |
|  |  | 3.0 | Solyc03g111640.3.1 | Programmed cell death 4 |
|  |  | 3.0 | Solyc06g051930.3.1 | pyruvate kinase superfamily protein |
| Sly-MIR171b | AAUUGAGCCGUGCCAAUAUCA | 1.5 | Solyc08g078800.1.1 | Transcription factor GRAS |
|  |  | 1.5 | Solyc01g090950.3.1 | Transcription factor GRAS |
|  |  | 2.5 | Solyc12g009790.2.1 | Calcium-dependent lipid-binding |
|  |  | 3.0 | Solyc12g009700.2.1 | Calcium-dependent lipid-binding |
|  |  | 2.5 | Solyc02g086540.2.1 | Core-2/I-branching beta-1,6-N-acetylglucosaminyltransferase family protein |
|  |  | 3.0 | Solyc08g081890.3.1 | ABC transporter family protein |
|  |  | 3.0 | Solyc09g011070.1.1 | clade XI lectin receptor kinase |
| Sly-MIR171c | UUGAUUGAGCCGCGCCAAUAU | 0.0 | Solyc08g078800.1.1 | Transcription factor GRAS |
|  |  | 1.0 | Solyc01g090950.3.1 | Transcription factor GRAS |
|  |  | 3.0 | Solyc09g008240.3.1 | ABC transporter B family protein |
| Sly-MIR171d | UUGAGCCGCGCCAAUAUCACU | 0.0 | Solyc08g078800.1.1 | Transcription factor GRAS |
|  |  | 1.0 | Solyc01g090950.3.1 | Transcription factor GRAS |
|  |  | 2.0 | Solyc11g013150.1.1 | Transcription factor GRAS |
|  |  | 3.0 | Solyc09g090090.2.1 | Phosphoenolpyruvate carboxylase kinase 2 |
|  |  | 3.0 | Solyc04g080970.3.1 | Lateral root primordium protein-related |
| Sly-MIR171f | UGAUUGAGCCGUGUCAAUAUC | 1.0 | Solyc08g078800.1.1 | Transcription factor GRAS |
|  |  | 1.0 | Solyc01g090950.3.1 | Transcription factor GRAS |
|  |  | 3.0 | Solyc02g085600.1.1 | Transcription factor GRAS |
|  |  | 3.0 | Solyc08g081890.3.1 | ABC transporter family protein |
|  |  | 3.0 | Solyc07g020910.2.1 | Sister chromatid cohesion protein PDS5 like B-B |
|  |  | 3.0 | Solyc06g051930.3.1 | pyruvate kinase superfamily protein |
| Sly-MIR171j | UGAGCCGAACCAAUAUCACUC | 0.0 | Solyc11g013150.1.1 | Transcription factor GRAS |
|  |  | 1.5 | Solyc08g069180.3.1 | Kinase |
|  |  | 1.5 | Solyc07g063860.3.1 | transmembrane protein |
|  |  | 2.0 | Solyc12g042177.1.1 | ADP-ribosylation factor GTPase-activating protein |
|  |  | 3.0 | Solyc07g054200.3.1 | pre-mRNA splicing factor-like protein |
|  |  | 3.0 | Solyc02g085080.3.1 | Transmembrane protein 53 |
|  |  | 3.0 | Solyc09g015520.3.1 | Leucine-rich receptor-like protein kinase family protein |
| Sly-MIR171k | UUGAGCCGCGCCAAUAUCAUU | 0.0 | Solyc08g078800.1.1 | Transcription factor GRAS |
|  |  | 1.0 | Solyc01g090950.3.1 | Transcription factor GRAS |
|  |  | 2.0 | Solyc11g013150.1.1 | Transcription factor GRAS |
|  |  | 3.0 | Solyc09g090090.2.1 | Phosphoenolpyruvate carboxylase kinase 2 |
|  |  | 3.0 | Solyc04g080970.3.1 | Lateral root primordium protein-related, putative |
